# Supplementary material for: A set of multi-entry identification keys to African frugivorous flies (Diptera, Tephritidae)
Source: Zookeys. 2014 Jul 24;(428):97–108. doi: 10.3897/zookeys.428.7366 (PMC4143993; doi:10.3897/zookeys.428.7366)
Supplement: Supplementary material 6 — Key to Ceratitis [file zookeys-428-097-s006.zip › SF6_ZooKeys_key to Ceratitis/key/SF6_ZooKeys_key to Ceratitis/Media/Html/Ceratitis penicillata .htm]

Ceratitis (Pterandrus) penicillata Bigot


***Ceratitis*** **(*Pterandrus*) *penicillata*
Bigot**

*Ceratitis* ? *penicillatus* Bigot, 1891: 381.

Body
length: 4.40 (3.90-5.15) mm; wing length: 4.78 (4.45-5.25) mm.

Male

Head: Antenna yellowish orange, First flagellomere brown;
twice or three times as long as pedicel. Arista with moderately long rays;
ventral rays shorter and sparser than dorsal rays, especially basally. Frons
yellow, sometimes pale yellow; darker patches on ventral half, especially at
frontofacial angle; with short scattered setulae largely of same color as frons
(occasionally darker). Frontal setae well developed. Face pale, median part
with darker yellow to brown patch. Genal seta and setulae dark, well developed.

Thorax:
Postpronotal lobe white, without spot. Scutal pattern: ground color dark gray;
with streaks and darker markings but without distinct spots except prescutellar
white separate markings, with pale area in between. Scapular setae dark.
Scutellum white, basally without spots, apically with three separate black
spots, extending to basal half. Anepisternum along ventral margin only brown;
setulae pale.

Legs:
Yellow except where otherwise noted; setation typical for subgenus, mixed pale
and dark. Foreleg: femur with dorsal part brownish colored anteriorly and
posteriorly; posteriorly no bush, only dispersed setulae; ventral setae black.
Midleg: femur anteriorly with large brown patch in median part, basally with
few dispersed long pale setulae; tibia moderately broadened and anteriorly
brownish black on distal half (except along dorsal row of feathering where
yellow covered by short dark setulae), with black feathering ventrally along
distal half, dorsally slightly over distal half. Hindleg: femur brown on dorsal
half; at apical 0.25 with longer setulae dorsally and ventrally.

Wing:
bands yellowish brown to brown. Marginal band forming continuous band with
anterior part of discal band; cubital band free; medial band absent (in rare
cases a weak and incomplete band is present); crossvein R-M proximal to middle
of discal cell. Apex of vein R_1_ distal to level of crossvein R-M.
Orientation crossvein DM-Cu variable.

Abdomen:
Mostly yellow. Tergites 2 and 4 across posterior half with pale gray band.
Tergite 3 brown on posterior half. Tergites 4 and 5 along anterior margin
yellowish brown, latter posteriorly narrowly brown. Male epandrium in lateral
view with lateral surstylus long and slender; posterior lobe straight and
elongate.

Female

As
male except for the following characters. First flagellomere darker but not
brown. Face without darker coloration in median part. Crossvein DM-Cu oblique
anterobasally. Medial band in one paratype of *fumitactus* weakly present.
Legs without feathering, femora yellowish brown; forefemur posteroventrally
with pale pilosity. Abdominal tergite 3 sometimes almost completely brown;
tergite 5 on anterior 0.33 yellowish brown to brown, posteriorly without
brownish margin. Oviscape slightly shorter than preabdomen. Aculeus at least
eight times longer than wide; tip with distinct apical indentation and lateral
margin weakly sinuous.

(Description
after De Meyer & Freidberg, 2006)
